# Efficacy Analysis of Combining Sintilimab with Neoadjuvant Chemotherapy in Treating Middle and Advanced Rectal Cancer Based on Big Data

**DOI:** 10.1155/2022/8675587

**Published:** 2022-09-16

**Authors:** Yifei Wang, Jiandong Fei, Yanan Zheng, Ping Li, Xiaodong Ren, Yongzhu An

**Affiliations:** ^1^Department of Gastrointestinal Surgery, The First Affiliated Hospital of Hebei North University, Zhangjiakou, 075000 Hebei, China; ^2^Central Laboratory, The First Affiliated Hospital of Hebei North University, Zhangjiakou, 075000 Hebei, China

## Abstract

**Objective:**

To analyze the efficacy of combining sintilimab with neoadjuvant chemotherapy in treating middle and advanced rectal cancer based on big data.

**Methods:**

According to the inclusion and exclusion criteria, 43 patients with middle and advanced rectal cancer, who were treated with sintilimab and neoadjuvant chemotherapy in General Surgery of the hospitals of Zhangjiakou city from January 2020 to January 2022, were selected for the retrospective study. The patients' short-term efficacy was scientifically evaluated, and the factors affecting efficacy and the correlation were analyzed.

**Results:**

Among the 43 enrolled patients, 30 of them had regional lymphatic metastasis but none had distant metastasis; most patients were at Broders II and TNM III, and all of them had adenocarcinoma; the total response rate was 69.77% (30 cases), with no grade IV and V adverse reactions; the patients were divided into the effective group and the ineffective group after treatment based on the evaluation results of short-term efficacy, and analysis of the relevant factors exposed in both groups revealed significant differences in age, tumor size, CEA, NLR value, PLR value, TNM stage, and presence of combined lymphatic metastasis between the two groups (*P* < 0.05); univariate analysis showed that tumor size, CEA, TNM stage, and combined lymphatic metastasis were the independent risk factors affecting the efficacy in patients with middle to advanced rectal cancer (*P* < 0.05); and through the Spearman correlation analysis of the above independent risk factors, it was further confirmed that tumor size, CEA, TNM stage, and combined lymphatic metastasis were negatively correlative with the efficacy of combining sintilimab with neoadjuvant chemotherapy in treating middle to advanced rectal cancer (*P* < 0.05).

**Conclusion:**

Combining sintilimab with neoadjuvant chemotherapy has good efficacy and safety profile, which is conducive to subsequent surgery; in contrast, larger tumor diameter, higher CEA level, higher TNM stage, and more serious lymphatic metastasis are all independent risk factors affecting treatment sensitivity and can lead to poor efficacy.

## 1. Introduction

Rectal cancer is one of the most common malignant tumors of the digestive tract. According to the 2020 global epidemiological statistics on cancer published by the International Agency for Research on Cancer (IARC), rectal cancer has become the 3rd most prevalent malignant tumor in men and the 2nd in women worldwide, with the highest incidence in developed countries such as North America, Australia, and New Zealand and 392,000 new cases in China in 2018. Its early symptoms are not obvious, and the incidence is relatively high in men aged 40-80 years. At present, surgical treatment is in the central position in rectal cancer treatment, because it provides patients at the early stage with long-term survival and those who can only receive limited treatment if they have local recurrence or distant metastasis with better survival in combination with chemoradiotherapy [1]. For patients with locally advanced rectal cancer, neoadjuvant chemotherapy can lower the tumor stage, and some reports have confirmed that the application of neoadjuvant chemotherapy before total mesorectal excision (TME) is the “gold standard” for the treatment of patients with stage II and III rectal cancer [2, 3]. In addition, some published works and reports also state that neoadjuvant chemotherapy has the potential to reduce local recurrence. In recent years, the studies of immune checkpoint inhibitors (ICIs) in various tumors are growing vigorously, and the results have shown that ICIs have good therapeutic effect. Immunotherapy is claimed to significantly improve the prognosis of colorectal cancer patients, and ICIs have also been recommended as a first-line option for advanced rectal cancer [4–7]. Related studies suggest that ICIs show good effects in the neoadjuvant treatment of resectable rectal cancer; meanwhile, ICI drugs also have a great potential in the comprehensive treatment decision-making of locally advanced and early rectal cancer. In 2022, sindilizumab as an innovative PD-1 inhibitor drug was successfully included in the CSCO guidelines for the clinical use of ICIs, achieving the breakthrough that all first-line therapies for five major tumors were included in the CSCO guidelines [8–10]. The use of immunotherapy in neoadjuvant treatment is mostly dependent on the safety and efficacy of the treatment, and it is still in the stage of active exploration from relevant data at home and abroad, with few clinical studies reported. Therefore, this article mainly collected middle and advanced rectal cancer patients treated with sindilizumab plus neoadjuvant chemotherapy in the General Surgery of the hospitals to carry out a retrospective study and to inquire about the clinicopathological factors related to the treatment efficacy, avoiding ineffectiveness or overtreatment, which is also beneficial to guide the preoperative treatment regimen and achieve the individualized treatment of middle and advanced rectal cancer.

## 2. Materials and Methods

### 2.1. Inclusion Criteria

(1) All patients were diagnosed with middle and advanced rectal cancer after CT, MRI, and pathological examination; (2) the TNM stages of the patients were IIc and III; (3) the ECOG score was less than 3 points; (4) the patients had basically normal routine blood test result, ECG, coagulation function, and liver and kidney function; (5) the patients failed in first-line oxaliplatin+targeted therapy chemotherapy and had not receive other PD-1 immunotherapy; (6) the patients did not have contraindications of sindilizumab and chemotherapy; (7) the patients received TME after neoadjuvant chemotherapy; and (8) the patients and their family members understood the study and signed the treatment consent and study consent.

### 2.2. Exclusion Criteria

Exclusion criteria are as follows: (1) complicated with other malignant tumors; (2) complicated with heart disease, severe hypertension, and other diseases of the cardiovascular system; (3) complicated with hematological system diseases and severe internal medicine diseases; (4) central nervous system metastases; (5) estimated survival less than 3 months; (6) concurrent acute infection, such as lung infection and urinary system infection; (7) pregnant or lactating women; and (8) low compliance with treatment and lost to follow-up.

### 2.3. Patient Screening

According to the inclusion and exclusion criteria, 43 patients with middle and advanced rectal cancer, who were treated with sintilimab and neoadjuvant chemotherapy in General Surgery of the hospitals of Zhangjiakou city from January 2020 to January 2022, were selected for the retrospective study; the study plan met the code of ethics and was reviewed and approved by the ethics committees of the hospitals of Zhangjiakou city.

### 2.4. Methods

#### 2.4.1. Sintilimab

Patients were treated with third-line and above anti-PD-1 mAb monotherapy or in combination with other agents, the single dosage of sintilimab injection (Tyvyt®) (specification: 100 mg; manufacturer: Innovent Biologics (Suzhou) Co., Ltd.; NMPA approval no. S20180016) was 200 mg, and it was administered once every 3 weeks until the downstaging effect and surgical resection indicators were met, so as to improve the rate of radical resection (R0 resection).

#### 2.4.2. Neoadjuvant Chemotherapy

XELOX scheme: from day 1 to day 14, 1000 mg/m^2^ of capecitabine was given twice daily; and at day 1, 130 mg/m^2^ of oxaliplatin was given. If a second course of chemotherapy was implemented, it should be started at day 22 of radiotherapy.

### 2.5. Observation Indicators

Clinical characteristics: the enrolled patients' clinical information including age (with 55 years old as the critical value), gender, smoking, Broders classification, ECOG score, and TNM stage was recorded.

Short-term efficacy: patients received reexamination after 4 weeks of treatment and were evaluated according to the WHO criteria for short-term objective response evaluation in solid tumors [11]. Complete disappearance of tumor lesions and maintenance for ≥4 weeks were considered complete remission (CR); ≥30% reduction in volume of tumor lesions compared with that before treatment and maintenance for ≥4 weeks represented partial remission (PR); <30% reduction in volume of tumor lesions compared with that before treatment or <20% increase represented stable disease (SD); and ≥20% increase in volume of tumor lesions compared with that before treatment or appearance of new lesions represented progressive disease (PD), with total response = CR + PR.

Adverse reactions: according to the Common Terminology Criteria for Adverse Events (CTCAE) v4.0 of the National Cancer Institute (NCI) [12], the adverse reactions were divided into grades I to V (grade I: mild adverse reaction, asymptomatic or mild symptoms, intervention not indicated; grade II: moderate adverse reactions, clinical symptoms that require intervention and may affect body function, but daily life will not be affected; grade III: severe adverse reactions, complicated symptoms that require active intervention and treatment; grade IV: life-threatening adverse reactions that may lead to disability and even organ damage or dysfunction; grade V: death).

Fasting venous blood was taken from all patients after treatment and tested for white blood cell count (normal range: 4.0–10.0 × 10^9^/L), neutrophils (normal range: 1.80–6.30 × 10^9^/L), lymphocytes (normal range: 0.8–4 × 10^9^/L), platelets (normal range: 100–300 × 10^9^/L), CEA (normal range: 3.5–5.0 ng/mL), CA199 (normal range: <37 IU/mL), NLR (normal range: 1–3), and PLR (normal range: 63.0–182.6).

The related factors, such as age (>55 years old), tumor size (≥3 cm), gender (male), distance between tumor and anal verge (≥5 cm), concurrent chemotherapy cycle (≥3 weeks), neoadjuvant chemotherapy cycle (≥2 weeks), white blood cell count, neutrophil, lymphocyte, platelet, CEA, CA199, NLR value, PLR value, TNM stage, and lymph node metastasis, were included in the single variate analysis of treatment effectiveness, and then, logistic regression analysis and Spearman correlation analysis were performed.

### 2.6. Statistical Processing

In this study, the data processing software was SPSS 22.0, which was mainly used to calculate the between-group differences of data; the picture drawing software was GraphPad Prism 7 (GraphPad Software, San Diego, USA); the items included were enumeration data and measurement data, which were expressed by [*n*(%)] and ($\bar{x}\pm s$), examined by the *X*^2^ test and *t*-test, and met normal distribution; and differences were considered statistically significant when *P* < 0.05.

## 3. Results

### 3.1. Clinical Characteristics

Among the 43 enrolled patients (aged 42 to 74 years) of the study, there were 28 males and 15 females, 30 patients had regional lymphatic metastasis, and none had distant metastasis; most patients were at Broders II and TMN III stage, all of them had adenocarcinoma. See Table 1 for statistical data.

### 3.2. Short-Term Efficacy

**Figure 1:**
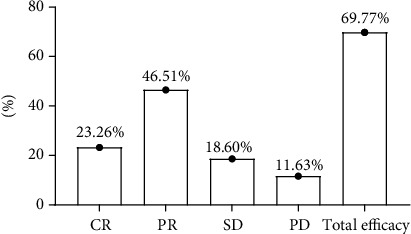
Evaluation results of patients' short-term efficacy.

All 43 patients finished sintilimab treatment, neoadjuvant chemotherapy, and surgical treatment and completed clinical efficacy evaluation. There were 10 CR cases, 20 PR cases, 8 SD cases, and 5 PD cases and a total of 30 effective cases. See Figure 1.

### 3.3. Adverse Reactions

After recording patients' adverse reactions, it was found that patients did not have grade IV and V adverse reactions, and the incidence rate of vomiting and nausea was the highest, followed by granulocytopenia and mucocutaneous damage. See Table 2.

### 3.4. Analysis of Factors Related to Treatment Effectiveness

The patients were divided into the effective group (*n* = 30) and the ineffective group (*n* = 30) after treatment based on the evaluation results of short-term efficacy, and analysis of the relevant factors exposed in both groups revealed significant differences in age, tumor size, CEA, NLR value, PLR value, TNM stage, and presence of combined lymphatic metastasis between the two groups (*P* < 0.05). See Table 3.

### 3.5. Logistic Regression Analysis

After including the single factors into logistic regression analysis, it was showed that tumor size, CEA, TNM stage, and lymphatic metastasis were independent risk factors affecting the efficacy of combining sintilimab with neoadjuvant chemotherapy in treating middle and advanced rectal cancer (*P* < 0.05). See Table 4.

### 3.6. Correlation Analysis

After performing Spearman correlation analysis of the above independent risk factors, it was further confirmed that tumor size, CEA, TNM stage, and combined lymphatic metastasis were negatively correlated with the efficacy of combining sintilimab with neoadjuvant chemotherapy in treating middle and advanced rectal cancer (*P* < 0.05). See Table 5.

## 4. Discussion

In recent years, neoadjuvant chemoradiotherapy has gradually walked into the popular field, and numerous previous studies have shown that the comprehensive treatment mode of neoadjuvant therapy combined with radical surgery has a relatively good effect on reducing the pathological stage of middle and advanced rectal cancer, lowering the rate of local recurrence, prolonging survival time, and improving the rate of anal conserving and the quality of life [13, 14]. Through preoperative neoadjuvant chemoradiotherapy, it is able to achieve postoperative pathological outcome as CR with low chance of recurrence in some patients; in addition, it can reduce the total number and positive rate of lymph nodes in postoperative specimens, which is beneficial to the improvement of the curative resection rate; and finally, neoadjuvant therapy reduces the tumor volume, increases the distance between the lower edge of the tumor and the anal verge, and increases the success rate of anal-conserving surgery. Meanwhile, concomitant with the continuous development of medical treatment, ICIs represented by anti-PD-1 mAb have become a research hotspot for cancer therapy, and a pan-tumor clinical trial study found that patients with mismatch repair dysfunction and high microsatellite instability might benefit from anti-PD-1 mAb therapy, but there is still insufficient evidence, and immunotherapy strategies for rectal cancer are under continuous exploration. The advent of sindilizumab marks the entry of antitumor immunotherapy in China into the innovation era. It is characterized by high affinity, long-lasting, stability and high target occupancy, and the objective response rate and disease control rate of immunotherapy using this drug for relapsed and refractory Hodgkin lymphoma are as good as those of innovative drugs of the international class [15, 16]. At present, radiotherapy combined with immunotherapy is the research hotspot of tumor regression, and the systemic immune response induced before surgery can make the body produce immune memory, while after surgery, patients cannot produce immune-mediated sustained antitumor effects due to tumor resection, so neoadjuvant chemotherapy combined with immunotherapy is also rational to a certain extent. Based on this, the efficacy of combining sindilizumab with neoadjuvant chemotherapy in treating middle and advanced rectal cancer was explored herein, and the related factors affecting efficacy were analyzed based on data.

The evaluation of clinical results of 43 patients revealed that there were 10 CR cases, 20 PR cases, 8 SD cases, and 5 PD cases, and the overall response rate of treatment was 69.77% (30 cases), which was higher compared with the previous results. On the one hand, the study result affirmed the efficacy of sindilizumab combined with neoadjuvant chemotherapy in patients with medium and advanced rectal cancer; on the other hand, because sindilizumab injection was officially marketed in mainland China only in 2019 and the duration of its clinical use for rectal cancer treatment is relatively short, so there are relatively few clinical cases. In addition, based on the study criteria, most of the enrolled patients were TNM stage III patients, some of them had regional lymph node metastasis, and none had distant metastasis, and therefore, the specificity of case screening may have some influence on the overall efficacy statistics. Based on the statistics of adverse reactions in patients, no grade IV and V adverse reactions occurred in all patients, and the incidence of nausea and vomiting was the highest, followed by granulocytopenia and mucocutaneous damage, which was close to most of the previous similar reported data [17, 18]. The overall incidence of adverse reactions was not low but was within an acceptable range from the point of view of clinical treatment. The patients were divided into the effective group and the ineffective group after treatment based on the evaluation results of short-term efficacy, and analysis of the relevant factors exposed in both groups revealed significant differences in age, tumor size, CEA, NLR value, PLR value, TNM stage, and presence of combined lymphatic metastasis between the two groups (*P* < 0.05); after including the single factors into logistic regression analysis, it was showed that tumor size, CEA, TNM stage, and combined lymphatic metastasis were the independent risk factors affecting the efficacy of combining sintilimab with neoadjuvant chemotherapy in treating middle and advanced rectal cancer (*P* < 0.05); and after performing Spearman correlation analysis of the above independent risk factors, it was further confirmed that tumor size, CEA, TNM stage, and combined lymphatic metastasis were negatively correlative with the efficacy of combining sintilimab with neoadjuvant chemotherapy in treating middle and advanced rectal cancer (*P* < 0.05). In terms of TNM studies in malignant tumors, reports have confirmed that the later the T stage of the tumor, the poorer the efficacy [19–21]. As the course of the disease progresses, the depth of tumor infiltration increases, the blood supply is relatively insufficient, and the tumor cells suffer from poor nutrition and hypoxia, which reduce the treatment sensitivity.

The sensitivity of mAb therapy and chemotherapy is dependent not only on the biological characteristics of the tumor itself but also on the microenvironment in which it resides. In vivo studies have found that tumor cells dying after chemoradiotherapy are able to present tumor associated antigens to host immune cells, activating the body tumor response. Therefore, hematological indices can also be an influential factor in the evaluation of recurrence and prognosis. The results of available studies suggest that neutrophils, NLR, lymphocytes, platelets, and CEA have some correlation with the efficacy and clinicopathological characteristics of neoadjuvant chemoradiotherapy and are somewhat valuable in judging the prognosis [22, 23]. CEA is widely used to predict the efficacy of neoadjuvant chemoradiotherapy for rectal cancer, with demonstrated efficacy predictive value. The changes in NLR and PLR, novel systemic immune response indicators, are caused by the synergistic effect of neutrophils, platelets, and lymphocytes. Relevant studies have shown that preoperative NLR levels and tumor volume size in cancer patients have significant associations [24, 25]. In this study, in logistic regression analysis and correlation analysis, the difference of NLR and PLR was not found, so the reference and guidance value of their level changes for the treatment of rectal cancer with the combination of sindilizumab and neoadjuvant chemotherapy should be confirmed with large-sample and multicenter studies.

In conclusion, combining sintilimab with neoadjuvant chemotherapy has significant efficacy in treating middle and advanced rectal cancer and is safer, which is conducive to the subsequent surgery; and larger tumor diameter, higher CEA level, higher TNM stage, and more serious lymphatic metastasis are all independent risk factors affecting treatment sensitivity and can lead to poor efficacy.

## Figures and Tables

**Table 1:** Clinical characteristics of 43 patients (*n* = 43).

Clinical data	Number of cases	Proportion (%)
Age		
≤55 years	20	46.51
>55 years	23	53.49
Gender		
Male	28	65.12
Female	15	34.88
Smoking		
Yes	24	55.81
No	19	44.19
Broders grade		
I	4	9.30
II	31	72.09
III	8	18.60
ECOG score		
0-1 point	26	60.47
2 points	17	39.53
TNM stage		
IIc	13	30.23
III	30	69.77

**Table 2:** Evaluation results of patients' adverse reactions.

Adverse reactions	Grade I	Grade II	Grade III	Grade IV	Grade V	Total incidence rate [*n* (%)]
Nausea	17	2	0	0	0	19 (44.19)
Vomiting	15	3	1	0	0	19 (44.19)
Diarrhea	6	5	0	0	0	11 (25.58)
Leukopenia	8	4	0	0	0	12 (27.91)
Granulocytopenia	11	4	2	0	0	17 (39.53)
Thrombocytopenia	3	3	0	0	0	6 (13.95)
Mucocutaneous damage	10	4	1	0	0	15 (34.88)
Neurotoxicity	9	2	0	0	0	11 (25.58)
Peripheral phlebitis	4	1	0	0	0	5 (11.63)

**Table 3:** Analysis of factors related to treatment effectiveness.

Related factors	Ineffective group (*n* = 13)	Effective group (*n* = 30)	*X* ^2^/*t*	*P*
Age (>55 years)	10 (72.92)	13 (43.33)	4.113	0.043
Gender (male)	7 (53.85)	21 (70.00)	1.042	0.307
Tumor size (≥3 cm)	8 (61.54)	8 (26.67)	4.721	0.030
Distance between tumor and anal verge (≥5 cm)	7 (53.85)	16 (53.33)	1.042	0.307
Concurrent chemotherapy cycle (≥3 weeks)	6 (46.15)	11 (36.67)	0.342	0.559
Neoadjuvant chemotherapy cycle (≥2 weeks)	8 (61.54)	16 (53.33)	0.248	0.619
White blood cell count (×10^9^/L)	3.82 ± 0.55	4.03 ± 0.68	0.981	0.332
Neutrophil (×10^9^/L)	3.04 ± 1.01	3.10 ± 1.04	0.175	0.862
Lymphocyte (×10^9^/L)	0.90 ± 0.54	0.92 ± 0.49	0.119	0.906
Platelet (×10^9^/L)	216.94 ± 60.16	218.33 ± 68.73	0.063	0.950
CEA (ng/mL)	5.24 ± 1.48	2.64 ± 1.28	5.836	<0.001
CA199 (IU/mL)	30.31 ± 12.16	23.85 ± 11.95	1.575	0.123
NLR value	4.16 ± 0.62	3.61 ± 0.85	2.098	0.042
PLR value	284.03 ± 13.85	270.11 ± 13.74	3.044	0.004
TNM stage			4.488	0.034
IIc	1 (7.69)	12 (40.00)		
III	12 (92.31)	18 (60.00)		
Lymphatic metastasis	12 (92.31)	18 (60.00)	4.488	0.034

**Table 4:** Multivariate logistic regression analysis.

Variable	B	S.E.	Wals	df	Sig.	Exp (*B*)	95% C.I. of Exp (*B*)
Tumor size	-1.482	0.704	4.431	1	0.035	0.227	0.057-0.903
CEA (ng/mL)	-1.240	0.376	10.904	1	0.001	3.457	1.656-7.218
TNM stage	-2.079	1.106	3.538	1	0.006	0.125	0.014-1.091
Lymphatic metastasis	-2.079	1.106	3.538	1	0.006	0.125	0.014-1.091

**Table 5:** Spearman correlation analysis.

Variable	*N*	Correlative coefficient	Significance
Tumor size	43	-0.331^∗^	0.030
CEA (ng/mL)	43	-0.664^∗∗^	<0.001
TNM stage	43	-0.323^∗^	0.035
Lymphatic metastasis	43	-0.323^∗^	0.035

Note: ∗ indicated significant correlation at the 0.05 level (two-sided); ∗∗ indicated a significant correlation at the 0.01 level (two-sided).

## Data Availability

Data to support the findings of this study is available on reasonable request from the corresponding author.
